# Supplementary material for: The Preclinical Evaluation of a Second-Generation Antivenom for Treating Snake Envenoming in India
Source: Toxins (Basel). 2022 Feb 24;14(3):168. doi: 10.3390/toxins14030168 (PMC8950585; doi:10.3390/toxins14030168)
Supplement: Supplementary file 1 [file toxins-14-00168-s001.zip › toxins-1523058-SI.pdf]

Supplementary Materials: The Preclinical Evaluation of a Second-Generation Antivenom for Treating Snake Envenoming in India

Saurabh Attarde, Ashwin Iyer, Suyog Khochare, Umesh Shaligram, Mayur Vikharankar and Kartik Sunagar *

Table S1. List of proteins identified in SIIPL-01 antivenom.

Sr. No.	Accession	-10lgP	Coverage (%)	#Peptides	#Unique	Avg. Mass	Protein family	Description
1	5DBY	360.42	48	54	54	65.526	Albumin	Chain A Serum albumin
2	XP_001499173.3	209.25	7	11	11	164.038	alpha macroglobin	alpha-2-macroglobulin [Equus caballus]
3	XP_014596182.1	87.35	3	3	3	163.134	alpha macroglobin	alpha-2-macroglobulin isoform X2 [Equus caballus]
4	XP_005607860.2	133.35	6	3	3	104.854	Fibrinogen	fibrinogen alpha chain [Equus caballus]
5	XP_003364583.1	123.12	7	4	4	56.25	Fibrinogen	fibrinogen beta chain [Equus caballus]
6	XP_023498059.1	179.5	7	10	10	239.429	Fibronectin	fibronectin isoform X12 [Equus caballus]
7	XP_001497860.1	186.65	33	11	11	38.466	Haptoglobin	haptoglobin [Equus caballus]
8	AAS18414.1	281.29	36	17	3	35.721	IgG	immunoglobulin gamma 7 heavy chain partial [Equus caballus]
9	CAC86340.1	269.95	38	21	5	35.885	IgG	immunoglobulin gamma 5 heavy chain constant region partial [Equus caballus]
10	AAS18415.1	265.18	34	15	1	35.72	IgG	immunoglobulin gamma 4 heavy chain partial [Equus caballus]
11	CAC86341.1	244.65	20	8	6	35.94	IgG	immunoglobulin gamma 6 heavy chain constant region partial [Equus caballus]
12	AAG01011.1	224.18	25	17	2	46.934	IgG	immunoglobulin G heavy chain partial [Equus caballus]
13	NP_001271464.1	180.76	28	6	6	17.852	IgG	immunoglobulin J chain precursor [Equus caballus]
14	CAC44760.1	158.85	19	6	3	37.424	IgG	immunoglobulin gamma 1 heavy chain constant region partial [Equus caballus]
15	CAC86339.1	145.95	16	7	5	38.651	IgG	immunoglobulin gamma 3 heavy chain constant region partial [Equus caballus]
16	XP_023507318.1	110.23	14	4	4	54.553	IgG	alpha-1B-glycoprotein [Equus caballus]

Toxins **2022**, *14*, 168. https://doi.org/10.3390/toxins14030168 www.mdpi.com/journal/toxins

17	AIY24824.1	107.66	17	2	1	23.373	IgG	immunoglobulin lambda light chain variable region partial [Equus caballus]
18	AAU09792.1	79.26	6	3	3	49.438	IgG	immunoglobulin mu heavy chain constant chain secreted form partial [Equus caballus]
19	AAP80145.1	64.77	6	1	1	37.039	IgG	immunoglobulin alpha constant heavy chain partial [Equus caballus]
20	ADK09620.1	58.61	6	1	1	19.901	IgG	immunoglobulin kappa light chain V-J region partial [Equus caballus]
21	ARU82570.1	47.66	11	1	1	14.452	IgG	immunoglobulin mu heavy chain partial [Equus caballus]
22	XP_014593633.1	125.5	3	2	2	82.01	Plasminogen	plasminogen isoform X2 [Equus caballus]
23	XP_001490892.4	195.44	9	7	7	70.229	Prothrombin	prothrombin [Equus caballus]
24	BAG69594.1	128.68	11	6	1	46.976	Serpin	alpha-1-antitrypsin [Equus caballus]
25	BAG69586.1	106.02	13	4	1	46.941	Serpin	alpha-1-antitrypsin [Equus caballus]
26	XP_014594947.1	104.68	3	2	2	52.37	Serpin	antithrombin-III [Equus caballus]
27	XP_001504386.3	38.27	2	1	1	54.63	Serpin	alpha-2-antiplasmin [Equus caballus]

Table S2. List of proteins identified in Virchow antivenom.

Sr. No.	Accession	-10lgP	Coverage (%)	#Peptides	#Unique	Avg. Mass	Protein family	Description
1	5DBY	287.55	19	16	16	65.526	Albumin	Chain A Serum albumin
2	XP_001488384.1	164.58	16	5	5	39.01	alpha macroglobulin	protein AMBP [Equus caballus]
3	XP_005607860.2	84.61	2	1	1	104.854	Fibrinogen	fibrinogen alpha chain [Equus caballus]
4	XP_023498059.1	100.07	1	2	2	239.429	Fibronectin	fibronectin isoform X12 [Equus caballus]
5	CAC86340.1	311.86	27	20	8	35.885	IgG	immunoglobulin gamma 5 heavy chain constant region partial [Equus caballus]
6	AAS18414.1	251.46	27	13	8	35.721	IgG	immunoglobulin gamma 7 heavy chain partial [Equus caballus]
7	AAU09792.1	228.94	27	13	12	49.438	IgG	immunoglobulin mu heavy chain constant chain secreted form partial [Equus caballus]
8	ARU82995.1	220.77	41	8	1	18.071	IgG	immunoglobulin mu heavy chain G partial [Equus caballus]
9	CAC44760.1	201.89	20	9	3	37.424	IgG	immunoglobulin gamma 1 heavy chain constant region partial [Equus caballus]
10	AAS18415.1	201.11	27	9	4	35.72	IgG	immunoglobulin gamma 4 heavy chain partial [Equus caballus]
11	ADK09716.1	188.87	21	7	1	22.777	IgG	immunoglobulin lambda light chain V-J region partial [Equus caballus]
12	XP_001499728.2	181.97	33	8	8	38.524	IgG	beta-2-glycoprotein 1 [Equus caballus]
13	AIY24807.1	180.03	23	8	1	23.315	IgG	immunoglobulin lambda light chain variable region partial [Equus caballus]
14	CAA53284.1	170.92	20	3	3	25.251	IgG	immunoglobulin kappa light chain [Equus caballus]
15	ADK09715.1	167.82	20	5	1	22.952	IgG	immunoglobulin lambda light chain V-J region partial [Equus caballus]
16	NP_001271464.1	165.74	30	6	6	17.852	IgG	immunoglobulin J chain precursor [Equus caballus]
17	ADK09688.1	165.46	21	5	1	21.392	IgG	immunoglobulin lambda light chain V-J region partial [Equus caballus]

18	XP_005601929.1	158.6	24	6	6	38.724	IgG	alpha-2-HS-glycoprotein [Equus caballus]
19	AIY24773.1	152.53	23	5	1	23.199	IgG	immunoglobulin lambda light chain variable region partial [Equus caballus]
20	AMR08103.1	143.17	21	4	1	12.665	IgG	immunoglobulin lambda light chain variable region partial [Equus caballus]
21	AMR08194.1	133.37	12	4	2	12.701	IgG	immunoglobulin lambda light chain variable region partial [Equus caballus]
22	AMR08187.1	116.95	19	3	1	12.031	IgG	immunoglobulin lambda light chain variable region partial [Equus caballus]
23	AMR08155.1	112.35	21	3	2	12.55	IgG	immunoglobulin lambda light chain variable region partial [Equus caballus]
24	AAP80145.1	108.79	14	4	4	37.039	IgG	immunoglobulin alpha constant heavy chain partial [Equus caballus]
25	AIM46367.1	106.49	12	3	1	12.79	IgG	immunoglobulin lambda light chain variable region partial [Equus caballus]
26	CAC44761.1	103.42	4	2	2	37.494	IgG	immunoglobulin gamma 2 heavy chain constant region partial [Equus caballus]
27	AMR08113.1	94.51	12	3	1	12.936	IgG	immunoglobulin lambda light chain variable region partial [Equus caballus]
28	ADK09861.1	86.65	12	2	1	21.135	IgG	immunoglobulin lambda light chain V-J region partial [Equus caballus]
29	ADK09588.1	84.46	10	1	1	19.094	IgG	immunoglobulin kappa light chain V-J region partial [Equus caballus]
30	AMR08131.1	80.68	24	3	1	12.781	IgG	immunoglobulin lambda light chain variable region partial [Equus caballus]
31	BAG69594.1	115.72	15	4	2	46.976	Serpin	alpha-1-antitrypsin [Equus caballus]
32	BAG69590.1	105.17	12	3	1	46.768	Serpin	alpha-1-antitrypsin [Equus caballus]

Table S3. List of proteins identified in Premium serums antivenom.

Sr. No.	Accession	-10lgP	Coverage (%)	#Peptides	#Unique	Avg. Mass	Protein family	Description
1	5DBY	518.6	77	149	149	65.526	Albumin	Chain A Serum albumin
2	XP_001499173.3	299.01	19	32	27	164.038	alpha macroglobulin	alpha-2-macroglobulin [Equus caballus]
3	XP_001488384.1	276.46	36	17	17	39.01	alpha macroglobulin	protein AMBP [Equus caballus]
4	XP_014596182.1	179.32	7	10	5	163.134	alpha macroglobulin	alpha-2-macroglobulin isoform X2 [Equus caballus]
5	XP_014596181.1	179.32	7	10	5	164.842	alpha macroglobulin	alpha-2-macroglobulin isoform X1 [Equus caballus]
6	XP_005607860.2	286.44	15	17	17	104.854	Fibrinogen	fibrinogen alpha chain [Equus caballus]
7	XP_003364583.1	278.55	25	15	15	56.25	Fibrinogen	fibrinogen beta chain [Equus caballus]
8	XP_001914833.2	172.91	15	7	7	49.796	Fibrinogen	fibrinogen gamma chain [Equus caballus]
9	XP_023498055.1	309.89	15	29	29	249.914	Fibronectin	fibronectin isoform X9 [Equus caballus]
10	XP_001497860.1	293.15	48	27	27	38.466	Haptoglobin	haptoglobin [Equus caballus]
11	AAS18414.1	350.64	43	28	6	35.721	IgG	immunoglobulin gamma 7 heavy chain partial [Equus caballus]
12	CAC86340.1	350.18	64	35	7	35.885	IgG	immunoglobulin gamma 5 heavy chain constant region partial [Equus caballus]
13	AAS18415.1	346.98	43	28	6	35.72	IgG	immunoglobulin gamma 4 heavy chain partial [Equus caballus]
14	AAG01011.1	335.86	47	33	9	46.934	IgG	immunoglobulin G heavy chain partial [Equus caballus]
15	XP_023507318.1	311.07	37	27	27	54.553	IgG	alpha-1B-glycoprotein [Equus caballus]
16	NP_001271464.1	289.5	53	17	17	17.852	IgG	immunoglobulin J chain precursor [Equus caballus]
17	CAC86341.1	271.98	34	14	4	35.94	IgG	immunoglobulin gamma 6 heavy chain constant region partial [Equus caballus]
18	XP_005601929.1	262.04	26	14	14	38.724	IgG	alpha-2-HS-glycoprotein [Equus caballus]
19	CAC44760.1	248.25	26	15	8	37.424	IgG	immunoglobulin gamma 1 heavy chain constant region partial [Equus caballus]
20	AAU09792.1	235.72	21	13	12	49.438	IgG	immunoglobulin mu heavy chain constant chain secreted form partial [Equus caballus]

21	CAC44761.1	226.82	23	12	6	37.494	IgG	immunoglobulin gamma 2 heavy chain constant region partial [Equus caballus]
22	AAA50981.1	226.01	48	11	1	22.668	IgG	lambda-immunoglobulin partial [Equus caballus]
23	AIY24847.1	225.68	50	13	1	23.534	IgG	immunoglobulin lambda light chain variable region partial [Equus caballus]
24	CAC86339.1	220.74	28	15	4	38.651	IgG	immunoglobulin gamma 3 heavy chain constant region partial [Equus caballus]
25	AAA50976.1	203.54	49	11	1	21.984	IgG	lambda-immunoglobulin partial [Equus caballus]
26	CAA53284.1	173.95	37	5	3	25.251	IgG	immunoglobulin kappa light chain [Equus caballus]
27	AIY24838.1	139.47	22	6	1	23.213	IgG	immunoglobulin lambda light chain variable region partial [Equus caballus]
28	ADK09609.1	123.6	20	3	1	19.282	IgG	immunoglobulin kappa light chain V-J region partial [Equus caballus]
29	AAP80145.1	115.57	10	2	2	37.039	IgG	immunoglobulin alpha constant heavy chain partial [Equus caballus]
30	ADK09589.1	77.87	17	2	1	19.019	IgG	immunoglobulin kappa light chain V-J region partial [Equus caballus]
31	ADK09619.1	68.69	7	1	1	19.558	IgG	immunoglobulin kappa light chain V-J region partial [Equus caballus]
32	XP_014593633.1	215.62	15	9	9	82.01	Plasminogen	plasminogen isoform X2 [Equus caballus]
33	XP_001490892.4	319.17	21	20	20	70.229	Prothrombin	prothrombin [Equus caballus]
34	BAG69594.1	264.39	28	17	4	46.976	Serpin	alpha-1-antitrypsin [Equus caballus]
35	BAG69585.1	222.67	16	8	2	46.864	Serpin	alpha-1-antitrypsin [Equus caballus]

Raw MS/MS spectra were searched against the National Center for Biotechnology Information's (NCBI) non-redundant (nr) database (Equus caballus: 9796) using Peaks Studio X+ for the identification of proteins present in the antivenoms. The key results of these searches, including the accession numbers, -10lgP values, number of high confidence peptides, unique peptides, average molecular mass (kDa), protein family and description of protein of the matching NCBI entry are listed here.

Table S4. Toxicity profiles of medically relevant 'big four' snakes.

Name of sample	Venom Dose (μg)					Number of survivors					LD_{50} (μg/mouse)	LD_{50} (mg/kg)
N. naja	**8**	**10**	**12.5**	**15.63**	**19.53**	**5**	**4**	2	1	0	12.22<br>10.89- 13.71	0.61<br>0.54- 0.69
D. russelii	4	5.12	6.4	8	10	5	3	2	1	0	5.71<br>4.87- 6.68	0.285<br>0.25- 0.34
B. caeruleus	1.44	1.73	2.07	2.5	3	5	4	1	0	0	1.90<br>1.78- 2.02	0.095<br>0.089- 0.101
E. carinatus	8.33	10	12	14.4	17.28	5	4	1	0	0	10.95<br>10.26- 11.68	0.55<br>0.51- 0.58

The table indicates various dose groups, survival patterns and toxicities of 'big four' venoms from Tamil Nadu.

Table S5. Neutralisation potencies of second generation antivenom and Indian polyvalent antivenom.

Venom used	Antivenom used: Second generation antivenom manufactured by Serum Institute of India Pvt. Ltd. (Batch No. SIIPL-01)							
	**Challenge dose 5X LD_{50}**	**Amount of antivenom injected in the venom-antivenom mixture (µl)**				**ED_{50} (µl)**	**ED_{50} (µl antivenom/mg venom)**	**Potency of antivenom (mg/ml)**
N. naja	**3.05 mg/kg**	**32.94**	21.97	14.64	9.76	17.93<br>15.53 - 20.72	293.45<br>254.17 - 339.12	2.726<br>2.36 - 3.15
D. russelii	1.42 mg/kg	21.97	14.64	9.76	6.51	11.95<br>8.60 - 16.60	418.56<br>301.23 - 581.44	1.91<br>1.37 - 2.65
B. caeruleus	0.475 mg/kg	9.75	6.51	4.34	2.89	3.64<br>2.70 - 4.93	383.16<br>284.21 -518.95	2.08<br>1.54 - 2.81
E. carinatus	2.75 mg/kg	49.40	32.93	21.97	14.64	29.37<br>22.21 - 38.84	536.44<br>405.66 - 709.41	1.49<br>1.12 -1.97

Venom used	Antivenom used: Indian polyvalent antivenom manufactured by Virchow Biotech Private Ltd. (Batch No. PAS00718)							
	**Challenge dose**	**Amount of antivenom injected in the venom-antivenom mixture (µl)**				**ED_{50} (µl)**	**ED_{50} (µl antivenom/ mg venom)**	**Potency of antivenom (mg/ml)**
N. naja	3.05 mg/kg	111.11	73.97	49.40	32.94	51.41<br>41.30- 64.00	841.41<br>675.94 - 1047.46	0.951<br>0.764- 1.18
D. russelii	1.42 mg/kg	32.94	21.97	14.64	9.76	12.29<br>9.00- 16.61	430.47<br>315.24- 581.79	1.86<br>1.37- 2.51
B. caeruleus	0.475 mg/kg	21.97	14.64	9.76	6.51	11.95<br>10.34- 13.80	1257.89<br>1088.42 - 1452.63	0.636<br>0.551- 0.734
E. carinatus	2.75 mg/kg	32.94	21.97	14.64	9.76	17.93<br>15.52- 20.72	327.49<br>283.47- 378.45	2.45<br>2.12- 2.83

The above table shows the toxicity neutralisation potencies of second generation antivenom and commercial Indian antivenom manufactured by Serum Institute of India and Virchow Biotech Pvt. Ltd., respectively. The neutralisation potencies were estimated against medically relevant 'big four' snake venoms from Tamil Nadu with 5X LD_{50} as 'challenge dose' as noted in the table.

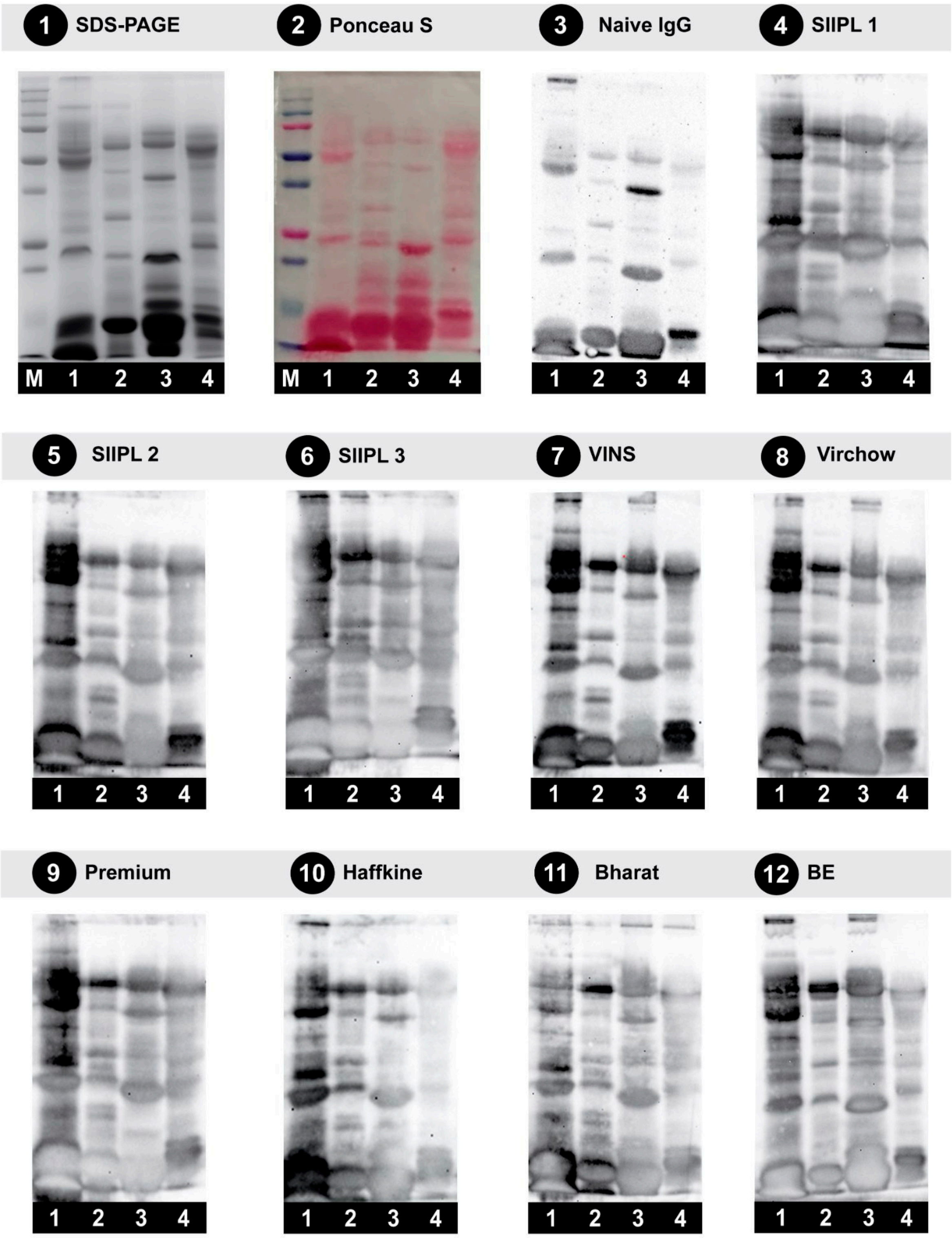

Figure S1. Binding affinities (western blots) of second-generation and conventional antivenoms against the ‘big four’ snake venoms from Tamil Nadu. 1: *N. naja*; 2: *D. russelii*; 3: *B. caeruleus*; 4: *E. carinatus*.

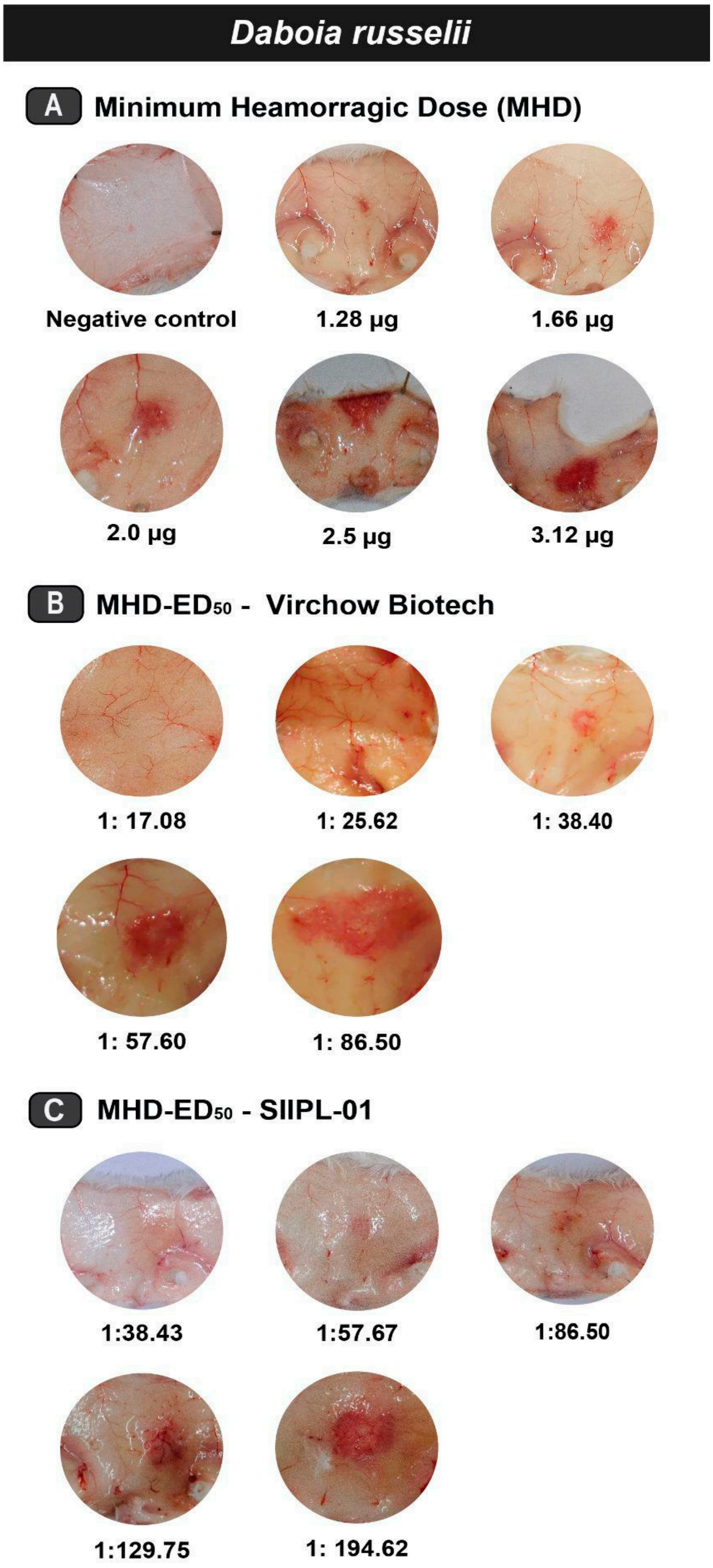

Figure S2. Hemorrhagic abilities of *D. russelii* venom and its neutralisation by antivenoms (A) MHD of *D. russelli* venom (B) MHD-ED$_{50}$ of Virchow antivenom (conventional product), (C) MHD-ED$_{50}$ of Serum Institute's antivenom (Second-generation product). Against *D. russelii* venom.

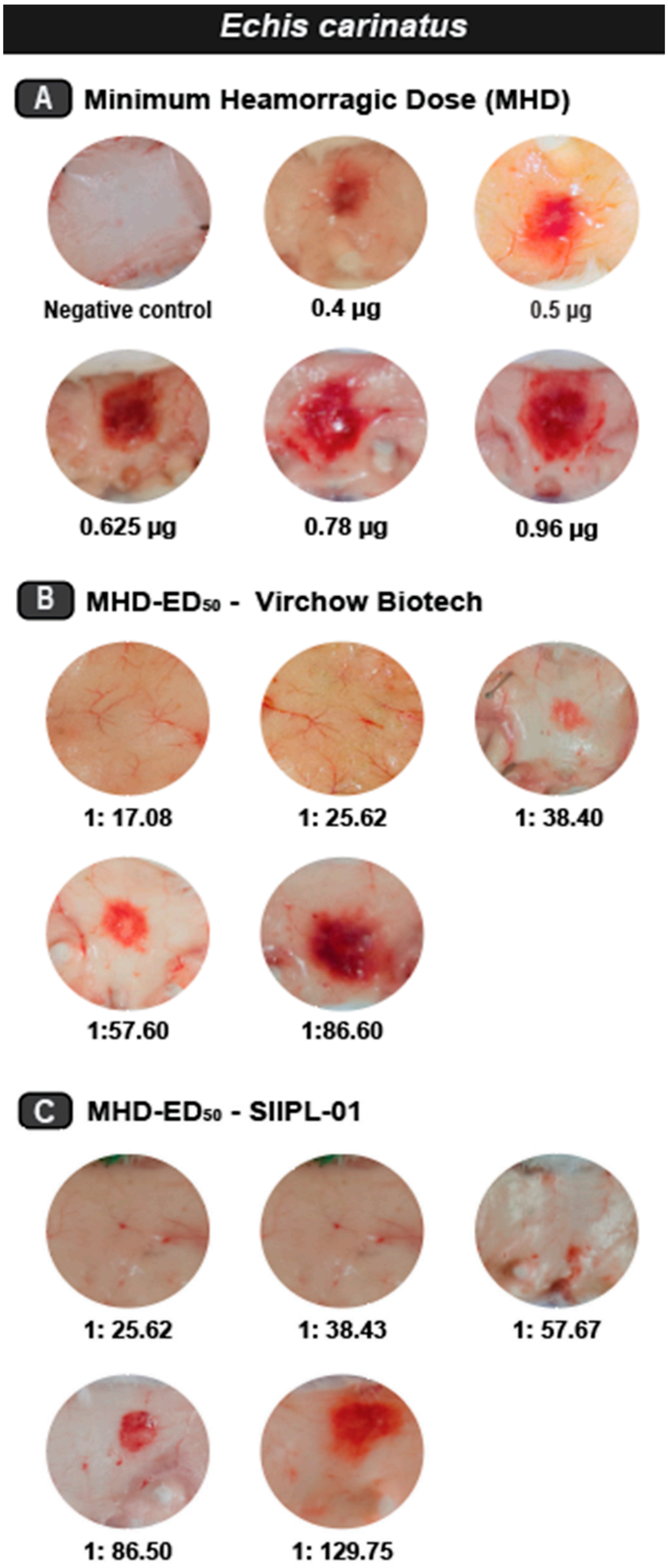

Figure S3. Hemorrhagic abilities of *E. carinatus* venom and its neutralisation by antivenoms (A) MHD of *D. russelii* venom and (B) MHD-ED_{50} of Virchow antivenom (conventional product), (C) MHD-ED_{50} of Serum Institute's antivenom (Second-generation product) against *E. carinatus* venom.

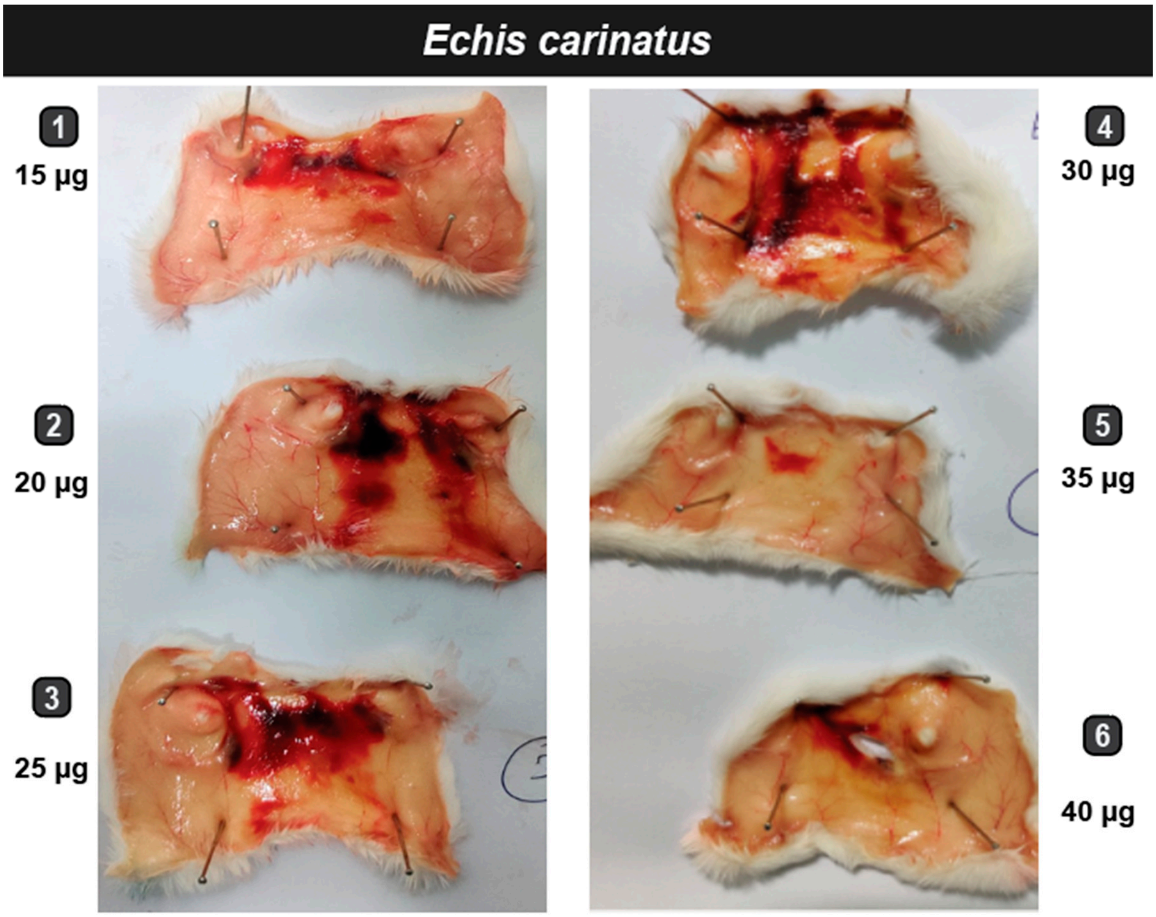

Figure S4. Lesions caused due to increasing concentration (15 to 40 µg) *E. carinatus* venom in MND experiment.

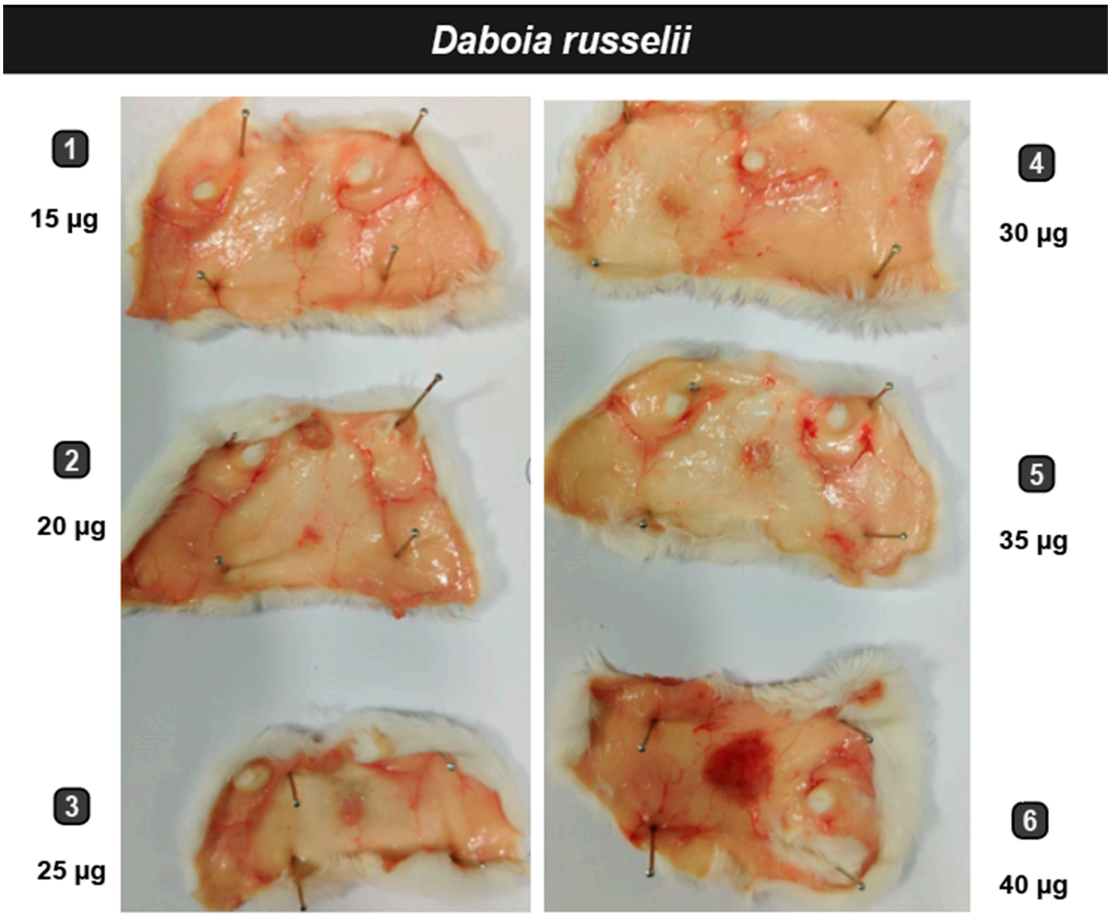

Figure S5. Lesions caused due to increasing concentration *D. russelii* venom (15 to 40 µg) in MND experiment.
